# Problematizing medical students with disabilities: A critical policy analysis

**DOI:** 10.15694/mep.2018.0000045.1

**Published:** 2018-02-26

**Authors:** Duncan Shrewsbury, Lise Mogensen, Wendy Hu

**Affiliations:** 1 Institute of Health and Society; 2 School of Medicine

**Keywords:** disability, policy analysis, accreditation, widening participation, competence

## Abstract

This article was migrated. The article was marked as recommended.

**Background:** Ensuring diversity, and that the medical profession is representative of the varied communities it serves is a worldwide equity concern. The widening participation movement in higher education aims to attract more students from non-traditional backgrounds into university. Yet, there is persistent under-representation of students with disabilities in medical education, and subsequently, the profession. The inclusion of these students is greatly influenced by the policies which regulate and accredit medical schools, which demand that educators consider students with disabilities as future doctors. While these policies may aim to promote inclusion, they may also have unintended consequences. In this paper we critically analyse key policies in undergraduate medical education to examine how disability in medical students is represented and problematized, and the educational implications of such representations.

**Method:** Key policies concerning medical school accreditation and educational standards from the General Medical Council (UK), Australian Medical Council (Australia) were selected for analysis. Carol Bacchi’s ‘What’s the Problem Represented to Be?’ six critical questions approach was applied to conduct a critical interpretive analysis of how disability is problematized in these policies.

**Findings:** Our analysis revealed a distinctive *construction of disability in medicine*, supported by themes of *containment of disability, disability and competence,* and *disability and risk.* Disability is framed as risk and potential educational burden for schools which must adapt practices to meet legal requirements. Risk is conceptualised as a quality of individuals, rather than being constructed through interactions between persons and environments. The ways in which disability is problematized relates to presuppositions which have the effect of restricting access for learners with disabilities.

**Conclusions:** The policies which regulate medical education can inadvertently limit inclusion of students with disabilities by being silent on the value of a diverse medical workforce. Bacchi’s six critical questions are an accessible and practical method for identifying the norms and assumptions which may impede change in educational policy and practice. By making visible these hidden suppositions and their consequences for learners, their impact may be ameliorated, and progress made.

## Background

Ensuring that the future medical workforce embraces diversity and can represent, as well as respect, the varied peoples it serves, is a global concern ( Moore, Sanders, & Higham, 2013). In higher education this concern is met through widening participation movements which have spread across education systems in the United Kingdom (UK), United States and Australia ( Moore et al., 2013; Rickinson, 2010; Shrewsbury, 2015; Zaxove, 2016). The policies and programs which underpin this movement aim to address the unequal representation of ‘non-traditional’ students. Typically, this refers to aspiring students from less affluent socio-economic backgrounds, but also extends to students with other under-represented characteristics, such as ethnic minority, and disability ( Moore et al., 2013). Disability can be defined in different ways; there is a chronic state of impairment (loss or difference of physiological, physical or psychological function) leading to disability (restricted social participation resulting from structural and social barriers) ( World Health Organization, 2001). In addition to equity considerations and the requirements of anti-discrimination legislation, it is also argued that medical students with disabilities, due to their personal experiences, bring positive attributes such as enhanced patient-centeredness, to their roles as future doctors ( Roberts, Butler, & Boursicot, 2004).

Yet, despite these compelling arguments and the mandatory requirements of accreditation standards and policies aimed at inclusion, it appears that the representation of disabled learners in medicine remains disproportionately low ( Rickinson, 2010; Shrewsbury, 2015). General community estimates suggest the prevalence of disability in working age adults to be 17% in the UK ( Scope., 2016) and 18.3% in Australia ( Australian Bureau of Statistics, 2016). In higher education it is estimated to be 7% in the UK (Higher Education Statistics Agency, 2015) and 5% in Australia ( Australian Disability Clearinghouse on Education and Training, 2015). In UK medical programs approximately 4.1% of students have a disability ( Shrewsbury, 2015). Data from an Australian university disability service suggests that less than 2% of medical students have registered a disability or chronic illness ( Director Disability Service Western Sydney University, 2016). Non-disclosure and under-reporting are likely, despite notable examples of individual students and programs ( Fitzsimons, Brookman, Arnholz, & Baker, 2016). Even so, it is likely that the prevalence of disability in medical students remains significantly below the goals of widening participation policies.

One reason for the persistence of under-representation is the challenge of balancing educational aims and ideals with responsibilities to patients and communities that the medical profession serves. As well as considering the functional ability of learners, educators must consider impairments that may impact on the ability to perform essential professional tasks, which may then affect the quality of care a patient receives ( General Medical Council, 2015a). Together with learners, the interests of stakeholders such as patients, employers, universities, the medical profession and the broader community must be taken into account when designing curricula and formulating policy.

**Figure 1.**
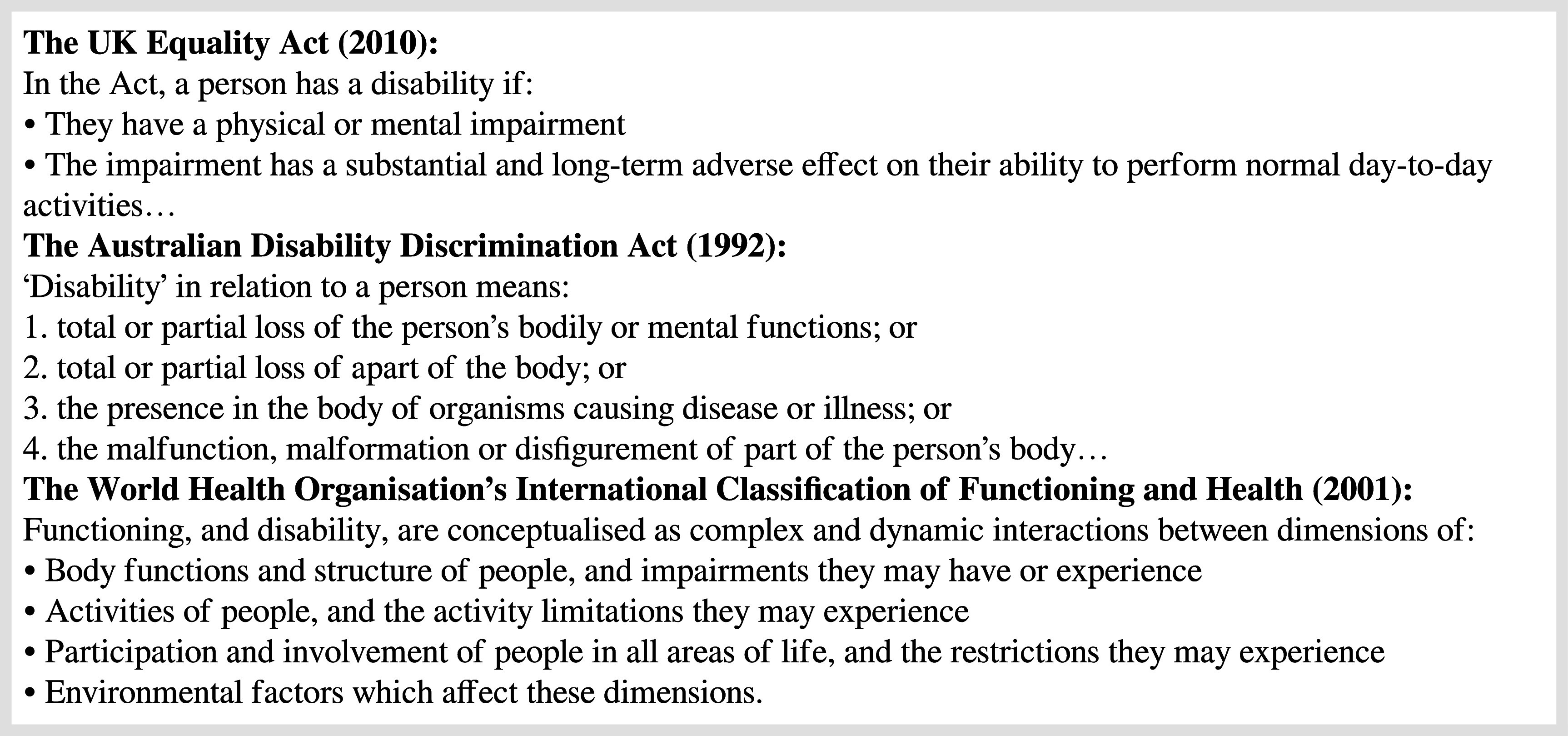
Disability as defined in UK (United Kingdon Government, 2010) and Australian ( Australian Government, 1992) law, and conceptualised in the World Health Organisation’s International Classification of Functioning and Health ( World Health Organization, 2001).

Adding to these complexities, disability and impairment can be understood from multiple perspectives within policy and practice ( World Health Organization, 2001). One is the biomedical model which focuses on pervasive deficits in mental or physical function, and their impact on norm-referenced abilities to perform tasks to a minimum expected level. This model is reflected in the national laws which govern disability policy ( Australian Government, 1992; UK Government, 2010). Counter to the view that disability is inherent in the individual, are social models which posit that disability is constructed when a social convention (e.g. expectations that buildings should be accessed by stairs) restricts participation (e.g. people who use wheelchairs). Such models suggest that disability is a construction leading to social exclusion and oppression ( Oliver, 1990; Shakespeare, 2013). There also are other, more nuanced perspectives which consider the interaction of a wide range of factors ( World Health Organization, 2001), which may be more acceptable as a means of understanding disability in medical practitioners ( Snashall, 2009) (see **Figure 1**). We therefore acknowledge that there is no ‘correct’ terminology in disability ( Rix, 2006), and follow the Person First convention by placing the inherent value of the individual before the disability ( World Health Organization, 2001).

Multiple perspectives, varied stakeholder interests, and persisting under-representation despite compelling arguments and policies to the contrary are features of ‘wicked problems’. This term was coined by Rittel and Webber to describe seemingly intractable issues in public policy planning and implementation ( Rittel & Webber, 1973). Framing the persistent underrepresentation of students with disabilities in medicine as a ‘wicked problem’ may assist to identify unrecognized barriers to participation and ways in which they may be addressed. We used this concept to frame our analysis of key policies which regulate medical education, with the aim of describing how the ‘problem’ of including students with disabilities is represented, and to explore how such representations may perpetuate the current situation.

## Theoretical Framework: What’s the Problem Represented to Be?

Carol Bacchi’s ‘What’s the problem represented to be?’ (WPR) approach to policy analysis ( Bacchi, 2012) provides a way to investigate how policy can fail to address complex, or wicked, problems ( Churchman, 1967). Her interpretive methodology centers on revealing the presuppositions on which a problem has been formulated ( Bacchi, 1999). These problem *representations* are important because they reflect how public issues are conceived and reproduced throughout society ( Churchman, 1967). In contrast to traditional policy analysis, WPR challenges the assumption that problems can be easily defined and are objective in nature ( Bacchi, 1999, 2009). The focus is on *problematizations,* rather than on problems, and on identifying the connections between what policies achieve - or do not achieve - and the assumptions upon which they are founded. The word ‘problematization’ may seem contentious in the context of learners with disabilities. However, we use the term without conferring judgement as to whether the problematization is negative (i.e. problematic) or positive.

**Figure 2.**
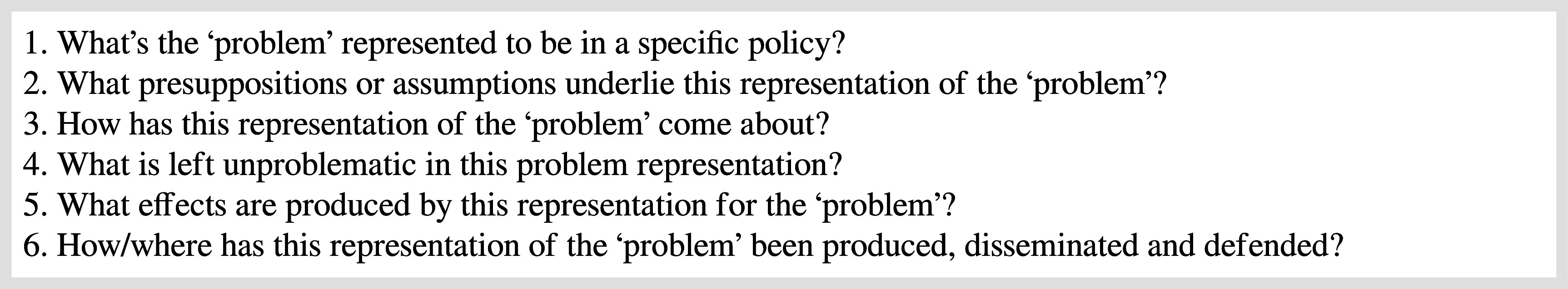
‘What’s the problem represented to be?’: 6 critical questions. ( Bacchi, 2012)

Bacchi proposes six questions with which policy analysts and researchers can critically interrogate policy (see **Figure 2**) ( Bacchi, 2009). The approach has provided greater clarity and assisted in advancing stalled debates about wicked policy issues such as gender inequality ( Bacchi, 1999, 2009), but to date has not been used in medical education. We therefore applied WPR’s critical questions to examine key policies in medical education and answer the questions:

• How is disability, and medical students with disability, problematized in medical education policies?
• What are the implications of these problematizations for inclusion of students with disabilities in medical education?

## Method

Using Bacchi’s WPR approach ( Bacchi, 2012) we analyzed and compared key policies that regulate and influence undergraduate medical education in Australia and the UK, with respect to students with disabilities. Taking a critical interpretive stance, we examined the ways in which disability is represented, and the educational implications of these constructions of disability within regulatory discourses.

### Data source

We chose Australian and UK policies because in these nations inclusive policies in education and employment have been recently strengthened as well as being the subject of public debate for time ( Oliver, 1990; Roberts et al., 2004). Commonalities in the history, structure and regulation of medical education also allow for a more nuanced and detailed analysis of assumptions and values, and where they may diverge. From the UK, we selected the General Medical Council’s (hereafter referred to as “GMC”) *Promoting excellence: standards for medical education and training* 2015( General Medical Council, 2015b), and from Australia, *Good Medical Practice* (“GMP Aust”) 2014 ( Medical Board of Australia, 2014), and *Standards for Assessment and Accreditation of Medical Schools* from the Australian Medical Council (“AMC”) 2010 ( Australian Medical Council, 2010). The GMC and AMC documents contain the standards that all medical schools must meet for accreditation in their respective countries, the GMP document outlines the principles of professional practice for all medical practitioners in Australia and is derived from the UK document of the same name (“GMP UK”) ( General Medical Council, 2013). The AMC and GMP documents cover the same scope of Australian educational practice as the GMC document for the UK.

These documents ( Australian Medical Council, 2010; General Medical Council, 2015b; Medical Board of Australia, 2014) are the key policies used to assure the quality of primary medical training in the UK and Australia. As well as describing the standards with which medical schools must comply, they also reflect community expectations about the obligations of the medical profession to provide competent and safe patient care. The policies regulate how medical education is developed and delivered within medical schools, and comply with relevant disability laws in the respective countries.

### Data analysis

Using WPR questions as an analytic lens ( Bacchi, 2009, 2012), we (the three authors) independently reviewed the text of the policies, developing the preliminary analysis through iterative discussion and written descriptions of preliminary themes. We then tested the emergent themes and findings by returning to the data. A constant comparative technique ( Glaser, Strauss, & Strutzel, 1968) was used throughout the analysis to compare Australia and UK documents and identify similarities and strong or common themes, or differences. These were then refined into the final themes and descriptors.

### Ethical approval

As the data was publicly available, no formal ethical approval was necessary. Ethical standards outlined by the British Educational Research Association (BERA) were observed (BERA, 2011).

### Researcher positioning

All three of us have experiences and/or responsibilities in supporting learners with disabilities and are active researchers in this field. DS is a medical doctor completing a PhD in special educational needs in medical education at a UK university, and has personal experience of a specific learning difficulty. LM has a background in occupational therapy, research interests in critical disability studies and has a formal role in supporting medical students with disabilities. WH is medically trained and is experiencing in developing, implementing and researching institutional policies in student support. Our analysis is therefore informed by our standpoints of enabling student participation and inclusion.

## Findings

Our interpretive analysis revealed greater similarities than differences between the two nations, so we present our findings as a combined narrative, informed by Bacchi’s critical questions.

Within an overarching theme concerning the *construction of disability in medical education*, we located three interrelated themes: *containment of disability, disability and competence,* and *disability and risk.* These themes are described below and illustrated with text (see **Appendix: Construction of Disability in Medical Education** for full text and document references, organized by theme).

## Containment of disability

Within the policies from both nations, disability was represented in restricted and limiting terms. In the GMP documents of both nations, disability is only named in relation to the practice of not discriminating against “disabled *patients*” [our italics] or those with disability (p.20) (see **Appendix ref: GMP Aust, GMP UK**). Disability is named in three GMC Requirements under Standard 3: *Supporting learners* and Standard 5: *Developing and implementing curricula and assessment standards* (see **Appendix ref: GMC R3.4, R3.5, R5.12**) and the one single AMC standard *Students* (see **Appendix ref: AMC Standard 7.3**). These references to disability, however, relate to compliance with anti-discrimination legislation, with the UK document citing the specific act. Guidance notes in the AMC standards state that medical schools “should not preclude *consideration*” of students with disability [our italics]. It states that there should be policies and procedures to inform students and to provide “appropriate support” and “reasonable accommodations” or adjustments to the program where they are deemed feasible, in keeping with “widening access schemes”, but disability itself is rarely specified or named (see **Appendix ref: AMCStandard 7.3 Notes**).

The GMC standards invoke the social model of disability by using the preferred language of UK disabled self-advocates, by referring to ‘disabled learners’ (see **Appendix ref: GMC R3.4, 5.2**). These standards are more rigorous by requiring a named contact person for reasonable adjustments, thus promoting institutional accountability. Nevertheless, none of the documents suggest that the inclusion of students with disabilities could enhance medical education and practice by being representative of the experiences and characteristics of the wider community.

Considering Bacchi’s question on presuppositions underlying these representations, students with disabilities are presumed to have additional, and it is implied, burdensome and potentially unreasonable needs, hence the need to limit responses to what is legally or minimally required. There is no suggestion that enhanced quality, or even excellence, could be achieved. Through a legalistic framing, disability is presumed to be dependent, monitored and contained, rather than being an opportunity for educational innovation.

This framing is further reinforced by the use of language; by adjusting for, or “accommodating” disability, the learner with a disability somehow needs to be *accommodated* rather than included and valued, and the system needs to withstand, tolerate and adapt. This language represents a power gradient, whereby the non-disabled *we*, have the power to bestow and gift accommodations to the disabled *you.* Bacchi’s question on the effects of this representation alludes to a systemic suppression of disability in medicine, so that doctors, who are providers of care cannot be simultaneously seen as recipients of care. The limited representation of doctors with disability in the Good Medical Practice documents suggests that disability is incommensurate with *good* medical practice.

## Disability and competence

The policies of both nations presume, and the UK document states, that good doctors can demonstrate a level of competence. One of the few explicit and specific references to disability and adjustments in the GMC concerns examination conditions to assess outcomes and competence (see **Appendix ref: GMC R5.12**). These standards require organizations to comply with the Equality Act 2010 and to make reasonable adjustments in order to help disabled learners meet the standards of competence, but the standards themselves cannot be changed, or by inference, weakened. In contrast there are no references to assessment support in the AMC policy. Rather, this document suggests that students with a disability are more likely to have problems with meeting the requirements of the course and of medical practice. The onus to meet competence standards is shifted to individuals by requiring schools to provide advice on the “demands” of the course and of the first postgraduate year, presumably so that would-be applicants can be deterred from applying. (see **Appendix ref: AMCStandard 7.3**)

In these sections, disability is located at the interface between individuals and an evaluation of desired levels of performance. Bacchi’s questions regarding presuppositions and how representations have come about highlight how this framing suggests that disability comes into being, or is constructed, at the point of assessment, when ensuring that standards of competence, which cannot be “changed” are met (see **Appendix ref: GMC R5.12**). The implication that competency standards could be compromised is emphasized over that of any intention to remove the barriers that impede success and achievement for disabled learners.

## Disability and risk

*Disability and risk* extends the idea of disability as a threat to competence, and thus to being a good doctor. The concept of impairment is invoked in these standards as part of health status and disability, but also when referring to concerning behaviors (e.g. drug or alcohol abuse) associated with unsafe or unprofessional practice. Thus, by association, ‘impairment’ suggests unsafe or unprofessional practice by individuals with disabilities, further problematizing disability as a threat to competence.

Elsewhere in the policies, disability is implicitly framed as a potential risk to patients and a threat to the profession. For example, the GMC document specifies the transitions to clinical placements and to graduate practice for additional support to students with disabilities, coinciding with increased exposure and responsibilities to patients and thus risk to patients. The reference is to ‘student needs’ (see **Appendix ref: GMC R3.5**), thus the focus remains on being needy, rather than on being potential contributors to patient care.

In relation to doctors’ health and professional behavior (See **Appendix ref: GMP Aust 9.2.7, 9.3.3**) impairment is invoked in this document through the professional obligation to report potentially sub-standard practice and to manage risks to patients. This repeated association of impairment with illness, incompetence or unprofessional behavior further problematizes disability as an undefined vulnerability of the individual (vulnerable to the effects of disablement) and to the system (vulnerability of the profession to the inclusion of learners with disabilities).

In considering Bacchi’s questions about the presuppositions leading to, and effects of, these representations, the notion of formal identification and mandatory reporting implies that disability is an either-or threshold state. However, disability is not explicitly defined in these documents, and only implicitly by reference to biomedical definitions in legislation (see **Figure 1**). Yet, once this ill-defined threshold is crossed, disability is problematized in limiting and potentially negative ways, compounding the idea of disability as a risk and a threat.

## Discussion

Our findings show how constructions of disability within key policies reveal how students with disabilities are seen within medical programs. Disability is largely contained and controlled through language and assumptions that focus on deficits and risks and on procedural requirements to satisfy legislation. We found that the concept of competence is strongly associated with being a good doctor, but disability is problematized as a threat to both assessing and reaching competence, and by implication, good doctoring. Impairment is associated with problematic practice, and not commensurate with competence, and therefore not to the professional image of the skilled practitioner.

Bacchi (2009) is clear that her approach serves to make explicit the presuppositions that underpin problem representations, and by doing so, to restart development on policy which has stalled. The absence of any reference to the value that disability may bring to medical education silences the widely accepted principles of inclusion and diversity in higher education. Our analysis supports the notion of widening participation as a wicked problem, with policy contributing to reproduction of the status quo through the effects of presuppositions. The gulf between policy aims and their enactment may also be due to policies being framed in ambiguous, unattainable and problematic ways. Our analysis identifies how norms and assumptions about disability as a biomedical deficit have led to policies which may deny the daily experience of learning with disability until there are points of assessment, or by taking a narrow view of risk to patient care.

Problematizations of disability in the examined policies constitute barriers for people living with a disability to enter and be valued in medical education. Drawing on the work of Bourdieu (1990), the construction of disability in medical education policy represents wider, unconscious, social mechanisms. For example, widening access initiatives may perversely increase recruitment of traditional students, rather than learners with disabilities, or other targeted groups ( Rainford, 2017). These contradictions reflect unresolved debates about representativeness in the profession, serving to further marginalize people with disabilities in society ( Albrecht, 2009).

Notions of thresholds and risk in these policies relate to an individual-focused, medical model view of disability, with the risk being located within the learner with disabilities. Such individualistic views neglect the wider social and cultural factors that contribute to the disabling system. Moreover, views which focus on individuals as threats to patient safety contradict current approaches to patient safety which recommend a systems evaluation approach to incidents and risk ( World Health Organisation, 2009). While the UK documents were more in alignment with a social model of disability, they were based on compliance with law, rather than any aspiration to widen inclusion. In the broader health education literature, there are approaches which describe the process of disablement and learning which could inform medical education practice ( Walker, Dearnley, Hargreaves, & Walker, 2013).

## Policy analysis in medical education

Given the social responsibilities of medical schools, it seems important to rigorously critique the policies which may drive, or limit, desired reform, and to identify ways forward by reframing debates so that they may lead to change. The WPR approach offers a systematic method to examine wicked problems in educational policies by: clarifying ambiguities; critically examining the ways in which policies achieve or limit outcomes; and by unpicking the presuppositions that perpetuate the problem. It is neutral in that it can be used to examine the implications of alternative representations. For example, different models of disability may be fit for purpose; from a human rights perspective, the social construction stance highlights oppressive social conventions that exclude people on the basis of impairment. From a quality of life perspective, the biomedical model identifies factors amenable to treatment or rehabilitation ( Shakespeare, 2013; World Health Organization, 2001).

WPR may also suggest responses by identifying alternative frames. For example, competence and risk can be re-conceptualized as the products of interactions between environments and systems which may fit poorly *with* individuals, not as inherent qualities *of* individuals. Such re-conceptualizations are already recognized within patient safety education ( World Health Organisation, 2009) but are not yet evident in the policies in our study. Without deprioritizing professional duties towards patients, we suggest that risk should be constructed as an interactional, context dependent process when considering the impact of learners on patient safety. For example, a doctor with physical disabilities who cannot perform cardio-pulmonary resuscitation could practice safely by being co-located with an assistant or clinician who can. Such arrangements will enable patients to benefit from the additional qualities and services that the doctor may bring. Focusing on individuals free of context may result in exclusionary practices which only serve to perpetuate resistance to inclusion of medical students with disabilities, to the detriment of patient care.

## Limitations

Interpretive analyses may be specific to, and therefore limited by, the prior knowledge, and ‘interpretive presuppositions’ of those conducting the analysis, and the selection of policies to analyze ( Yanow, 1999). In declaring our positions, we acknowledge that our interpretations may be unique. Rather than aiming to unearth absolute facts, interpretive policy analysis foregrounds the researchers’ backgrounds and collective experiences to interrogate policy ( Bacchi, 2009). Yanow (1999) argues that interpretive policy analysis rejects positivist presuppositions of knowledge in order to create opportunities for exploring meaning and values from different perspectives. Bacchi’s questions, by being neutral, invite alternative interpretations to ours, and renders our analysis explicit to readers. While such analyses may not result in immediate change or recommendations, they can push forward debates that are stuck or appear unsolvable.

Our selection of policies may limit transferability of our findings; we chose to analyze the 2010 version of the Australian standards as it contains more reference to disability through its guidance notes than the current 2012 version ( Australian Medical Council, 2012). The scope of the current 2012 version has not broadened, suggesting that structural constraints to participation persist. The selected policies continue to carry great influence in their nations of origin. There was more that was common, rather than divergent, between the two nations’ policies. This may be explained by their shared history in higher education, law and medicine, although the language in the more recent UK policy shows a stronger influence from the social model of disability. It may also be that the norms which govern disability are pervasive in nature. A larger selection of policies may have uncovered greater contradictions or evidence of reform, and we acknowledge the progress in UK medical education with the publication of GMC guides ( General Medical Council, 2015a) which provide recommendations for inclusive medical education, although they are yet to be strongly evidenced in policy and practice.

Our findings do however indicate that widening participation in medicine to students with disability remains a wicked problem. Advances may then be made by illuminating the hidden assumptions that restrain change and are continually reproduced in policy and practice. Educators may then examine how these assumptions are reflected in their teaching and learning programs, and researchers and policymakers to focus on the critical issues which then arise.

## Conclusions

There are compelling equity and patient care arguments to support widening participation in medical education. Ensuring that opportunities are accessible to otherwise qualified learners with disabilities is a social responsibility that has benefits for medical education and practice. Yet, discrepancies between these ideals and the actual representation of learners with disabilities persist. Our critical interpretive analysis of policies governing medical education in the UK and Australia alludes to how the language of policy may reflect norms and hidden assumptions. These in turn may lead to the unintended consequence of perpetuating barriers to learners with disabilities. It is therefore not surprising that such learners remain under-represented in medical education, and subsequently, the medical profession.

Bacchi’s questions offers an accessible and rigorous approach to analyze how policy may contradict stated aims, particularly for researchers new to interpretive policy analysis, and for educators and policymakers to use as a tool for evaluating their policies and programs. The ways in which wicked problems are conceived and perpetuated, and could potentially be addressed through medical education, policy and practice can thus be illuminated.

## Take Home Messages

• Despite moves to widen participation, students with disabilities remain under-represented in medical education
• Policies which govern medical education may unintentionally restrict inclusion of students with disabilities through limiting representations of disability and silence on the value of student diversity
• Bacchi’s What’s the Problem Represented to Be? is an accessible and rigorous method for identifying the assumptions that perpetuate wicked problems in medical education policy and practice

## Notes On Contributors

Duncan Shrewsbury is Senior Lecturer in Clinical Education and Primary Care, and academic GP registrar in the West Midlands, UK. He is completing his PhD at the Centre for Special Educational Needs and Disability at the University of Exeter.

Lise Mogensen is Lecturer in medical education, in the School of Medicine, Western Sydney University, Australia. Her professional background is in Occupational Therapy, she is the School Disability Adviser and is researching disability in the medical profession.

Wendy Hu is Professor of medical education, and Deputy Dean, School of Medicine, Western Sydney University, Australia. Her professional background is in General Practice and she leads an international research and training project in medical and health professional student support.

## Appendices

**Table:**

THE CONSTRUCTION OF DISABILITY IN MEDICAL EDUCATION Selected text from the Australian (GMP Aust, AMC) and UK (GMC, GMP UK) medical education policies, arranged by theme. Key words in analysis underlined.		
Themes	Australia	UK
**CONTAINMENT OF DISABILITY**	**GMP Aust 2.4.3 p6** *Upholding your duty to your patient and not discriminating on medically irrelevant grounds, including race, religion, sex, disability or other grounds, as described in anti-discrimination legislation* **AMC Standard 7.3 p29** *The school has policies on the admission of, and procedures for, the support of students with disabilities and students with infectious diseases, including blood-borne viruses.... The school has appropriate support for students with special support needs including those coming from under-represented groups or admitted through widening access schemes.* **AMC Standard 7.3 Notes p30** *Physical disability or infections should not preclude a student from consideration for admission to a medical school. Medical schools need to have in place policies and procedures on how students with such disabilities or infections will be accommodated in the medical course.....These policies and procedures should comply with relevant legislation and include provision of reasonable accommodation for disabled students. support and counselling services to deal with student illness, impairment and disability during the medical course.*	**GMP UK p20** *You must consider and respond to the needs of disabled patients and should make reasonable adjustments to your practice so they can receive care to meet their needs* **GMC Standard R3.4 p25** *Organisations must make reasonable adjustments for disabled learners, in line with the Equality Act 2010.* Organisations must make sure learners have access to information about reasonable adjustments, with named contacts.* **GMC Standard R5.12 p37** *Organisations must make reasonable adjustments to help disabled learners meet the standards of competence in line with the Equality Act 2010*
**DISABILITY AND COMPETENCE**	**AMC Standard 7.3 Notes p30** *... advice to potential students on both the demands of the medical course and the requirements of medical boards for provisional registration in the intern year.... The medical school publication that advises prospective students of the selection process for entry should outline the school’s policies with regard to disability and infection*	**GMC, Standard 5.2 p32** Medical school, and postgraduate curricula and assessments are *‘implemented so that doctors in training are able to do what is expected in Good Medical Practice.’* (refers to UK version) **GMC Standard R5.12 p37** *Organisations must make reasonable adjustments to help disabled learners meet the standards of competence in line with the Equality Act (2010), although the standards of competence themselves cannot be changed. Reasonable adjustments may be made to the way that the standards are assessed or performed (except where the method of performance is part of the competence to be attained), and to how curricula and clinical placements are delivered.*
**DISABILITY AND RISK**	**GMP Aust Principles 9.2.7 p22** *If you know or suspect that you have a health condition or impairment that could adversely affect your judgement, performance or your patient’s health....not relying on your own assessment of the risk you pose to patients...* **GMP Aust Principles 9.3.3 p22** *Encouraging a colleague (whom you are not treating) to seek appropriate help if you believe they may be ill and impaired. If you believe this impairment is putting patients at risk, notify the Medical Board of Australia.* **AMC Standard 7.3 Notes p31 4** *Because AMC accreditation of a medical school provides the graduates with eligibility for registration as medical practitioners, the AMC will enquire into the medical school’s mechanisms for dealing with students with impairment, and for addressing concerns about student health and behaviour issues with the medical board.*	**GMC p5** *Patient safety is at the core of these standards.... Where our standards previously focused on protecting patients from any risk posed by medical students and doctors in training, we will now make sure that education and training takes place where patients are safe, the care and experience of patients is good, and education and training are valued.* **GMC Standard R3.5 p25** *Learners must receive information and support to help them move between different stages of education and training. The needs of disabled learners must be considered, especially when they are moving from medical school to postgraduate training, and on clinical placements*

## References

[ref1] Albrecht G. (2009). The inclusion of disabled people in medical education.In Brosnan C. & Turner B. S. (Eds.), Handbook of the sociology of medical education. Routledge.

[ref2] Australian Bureau of Statistics. (2016). Disability, ageing and carers, Australia: Summary of findings, 2015. Retrieved from http://www.abs.gov.au/ausstats/abs@.nsf/mf/4430.0

[ref3] Australian Disability Clearinghouse on Education and Training. (2015). Statistics and research: access and participation. Retrieved from http://www.adcet.edu.au/inclusive-teaching/understanding-disability/research-and-statistics/

[ref4] Australian Government. (1992). Disability Discrimination Act, 1992. Retrieved from https://www.legislation.gov.au/Series/C2004A04426

[ref5] Australian Medical Council. (2010). Standards for the assessment and accreditation of medical schools by the Australian Medical Council 2010. Retrieved from http://www.amc.org.au/joomla-files/images/Medschool/accreditation-standards-medical-schools-2010.pdf

[ref6] Australian Medical Council. (2012). Standards for the assessment and accreditation of medical schools by the Australian Medical Council 2012. Retrieved from http://www.amc.org.au/joomla-files/images/Accreditation/FINAL-Standards-and-Graduate-Outcome-Statements-20-December-2012.pdf

[ref7] Bacchi C. (1999). Women, policy and politics: The construction of policy problems. Sage.

[ref8] Bacchi C. (2009). Analysing policy. Pearson Higher Education AU.

[ref9] Bacchi C. (2012). Introducing the “What’s the Problem Represented to be?” Approach. Engaging with Carol Bacchi: Strategic interventions and exchanges. 21–24.

[ref10] Bourdieu P. & Passeron J.-C. (1990). Reproduction in education, society and culture.(Vol.4): Sage.

[ref11] BERA Britsh Education Research Association (2011). Ethical guidelines for educational research. Retrieved from https://www.bera.ac.uk/wp-content/uploads/2014/02/BERA-Ethical-Guidelines-2011.pdf

[ref12] Churchman C. W. (1967). Guest editorial: Wicked problems. JSTOR.

[ref13] Director Disability Service Western Sydney University (2016). [Personal communication].

[ref14] Fitzsimons M. G. Brookman J. C. Arnholz S. H. & Baker K. (2016). Attention-deficit/hyperactivity disorder and successful completion of anesthesia residency: a case report. Academic Medicine. 91(2),210–214. 10.1097/ACM.0000000000000854 26244258

[ref15] General Medical Council. (2013). Good Medical Practice. Retrieved from https://www.gmc-uk.org/Good_medical_practice___English_1215.pdf_51527435.pdf

[ref16] General Medical Council . (2015a). Gateways to the professions: advising medical schools - encouraging disabled students. Retrieved from http://www.gmc-uk.org/Gateways_to_the_professions_Nov_2016.pdf_68375486.pdf

[ref17] General Medical Council . (2015b). Promoting excellence: standards for medical education and training. Retrieved from http://www.gmc-uk.org/Promoting_excellence_standards_for_medical_education_and_training_0715.pdf_61939165.pdf

[ref18] Glaser B. G. Strauss A. L. & Strutzel E. (1968). The discovery of grounded theory; strategies for qualitative research. Nursing research. 17(4),364. 10.1097/00006199-196807000-00014

[ref19] Higher Education Statistics Agency, H. E. S. (2015). Summary- UK Performance Indicators 2014/15. Retrieved from https://www.hesa.ac.uk/data-and-analysis/performance-indicators/summary

[ref20] Medical Board of Australia. (2014). Good Medical Practice: a code of conduct for doctors in Australia. Retrieved from http://www.medicalboard.gov.au/Codes-Guidelines-Policies.aspx 10.5694/mja13.11334 24528422

[ref21] Moore J. Sanders J. & Higham L. (2013). Literature review of research into widening participation to higher education: report to HEFCE and OFFA.

[ref22] Oliver M. (1990). The politics of disablement-New social movements The Politics of Disablement.(pp.112–131): Springer.

[ref23] Rainford J. (2017). Targeting of widening participation measures by elite institutions: widening access or simply aiding recruitment? Perspectives: Policy and Practice in Higher Education. 21(2-3),45–50. 10.1080/13603108.2016.1148645

[ref24] Rickinson M. (2010). Disability equality in higher education: a synthesis of research. Retrieved from EvidenceNet: Rix, J. (2006). Does it matter what we call them? Labelling people on the basis of notions of intellect. Ethical Space: The International Journal of Communication Ethics, 3(4), 22-28.

[ref25] Rittel H. W. & Webber M. M. (1973). Dilemmas in a general theory of planning. Policy sciences. 4(2),155–169. 10.1007/BF01405730

[ref26] Rix J. (2006). Does it matter what we call them? Labelling people on the basis of notions of intellect. Ethical Space: The International Journal of Communication Ethics. 3(4),22–28.

[ref27] Roberts T. Butler A. & Boursicot K. (2004). Disabled Students, Disabled Doctors-Time for a Change?: A Study of Different Societal Views of Disabled People’s Inclusion to the Study and Practice of Medicine: Higher Education Academy: Medicine, Dentistry and Veterinary Medicine.

[ref28] Scope. (2016). Disability facts and figures. Retrieved from http://www.scope.org.uk/media/disability-facts-figures

[ref29] Shakespeare T. (2013). Disability rights and wrongs revisited. Routledge.

[ref30] Shrewsbury D. (2015). Disability and participation in the professions: examples from higher and medical education Disability & Society. 30(1),87–100. 10.1080/09687599.2014.982785

[ref31] Snashall D. (2009). Doctors with disabilities: licensed to practise? Clinical medicine. 9(4),315–319. 10.7861/clinmedicine.9-4-315 19728501 PMC4952495

[ref32] United Kingdom Government. (2010). Equality Act. Retrieved from http://www.legislation.gov.uk/ukpga/2010/15/section/6

[ref33] Walker S. Dearnley C. Hargreaves J. & Walker E. A. (2013). Risk, fitness to practice, and disabled health care students. Journal of Psychological Issues in Organizational Culture. 3(S1),50–63. 10.1002/jpoc.21097

[ref34] World Health Organisation. (2009). WHO patient safety curriculum guide for medical schools. World Alliance for Patient Safety. Retrieved from http://www.who.int/patientsafety/information_centre/documents/who_ps_curriculum_summary.pdf?ua=1

[ref35] World Health Organization. (2001). International classification of functioning, disability and health (ICF). Retrieved from http://www.who.int/classifications/icf/en/

[ref36] Yanow D. (1999). Conducting interpretive policy analysis.(Vol.47). Thousand Oaks, CA: Sage Publications.

[ref37] Zazxove P. C. B. Moreland C Plegue MA Hoekstra A Ouellette A Sen A and Fetters MD. (2016). U.S. Medical schools’ compliance with the Americans with Disabilities Act: findings from a national study. Academic Medicine. 91(7). 10.1097/ACM.0000000000001087 26796093

