# Supplementary material for: Problematizing medical students with disabilities: A critical policy analysis
Source: MedEdPublish (2016). 2018 Feb 26;7:45. [Version 1] doi: 10.15694/mep.2018.0000045.1 (PMC10711971; doi:10.15694/mep.2018.0000045.1)
Supplement: Peer review history

# Reviewer report: Reviewer response for version 1

**DOI:** 10.21956/mep.19513.r28865

**Published:** 2019-02-27

**Authors:** Nicholas Lawson

**Affiliations:** 1 Georgetown University Law Center

This review has been migrated. The reviewer awarded 5 stars out of 5 This is a wonderful paper. What makes this paper so important is its focus on policies and structural stigma. Things are pretty bad for people with disabilities in society and the medical profession. It’s an era of discrimination de jure. One need not conduct a survey to assess public stigma related to disabilities when a quick look through a first world countries own official policies, widely disseminated, are extremely offensive and discriminatory. In addition, written policies like these can’t be denied—except by rejecting and excluding this manuscript from the mainstream medical education literature.The highlight and most moving part of the article was the policies themselves, in the Appendices. The GMP Australian Principles on disability and risk are inhuman, very disturbing, and very similar to the ones we have in the US. Are there any particular medical leaders who are responsible for these policies? Has anyone attempted to sue the Australian Medical Council? I am curious whether even more disturbing policies might be found in your countries’ equivalents to state physician health programs/medical boards/medical societies. In the US, disparaging rhetoric and discrimination on the basis of disability is often connected with wellness (well-being) programs, initiatives, and ideology, so I am curious if there are any such policies in your countries.In the US, there is an expectation, particularly in psychiatry, that persons with mental disabilities are supposed to take a lead role in guiding the profession and its priorities at all levels, but American Psychiatric Association (APA) heads would probably laugh at me if I suggested that that the next President of the APA should be an individual with a disclosed mental disorder or disability. Similarly, disability rights organizations certainly do not have the ears of the medical hierarchy or its priorities. Is this the same in your countries as well?

Reviewer Expertise:

NA

No decision status is available

# Reviewer report: Reviewer response for version 1

**DOI:** 10.21956/mep.19513.r28864

**Published:** 2018-04-15

**Authors:** Kirollos Nan

**Affiliations:** 1 Bond University

This review has been migrated. The reviewer awarded 4 stars out of 5 The analysis explores an ever-challenging topic that is unfortunately understated in its significance, especially in the context of current social trends. With diversity being radicalised within the medical field, the push for the inclusion of medical students with disabilities is an imperative issue. The analysis focuses on the policies that govern medical schools and how they are innately constructed to stunt the inclusion of such students. While there has been an active effort to ensure diversity, there is a claim that policies are constructed in such a way that undermines the ability of learners with a 'disability', questioning the notion that they may be unable to meet the needs of the community. The use of Bacchi's 6 questions helped highlight the restraints placed by the policies, emphasising the nature of the problem and the understatement of 'disabled' medical students. It truly helped pinpoint a key issue that stems within governing bodies and thus, identified a potential area that can be potentially targeted and resolved. It's quite remarkable to think that language can be a barrier to diversity.In contrast, as mentioned in the analysis, prior knowledge can greatly limit the qualitative analysis used in this article. Furthermore, it's quite difficult to accurately determine the exact consequences that these policy wordings may have on the under-representation of these students. Such a complex issue is likely to be multi-factorial which leads to another limitation, the lack of alternative factors that are leading to under representations of these students. The analysis may have fulfilled its intended outcomes, but it appeared to be one-dimensional.

Reviewer Expertise:

NA

No decision status is available

# Reviewer report: Reviewer response for version 1

**DOI:** 10.21956/mep.19513.r28862

**Published:** 2018-04-06

**Authors:** Anthony David.M

**Affiliations:** 1 Soho

This review has been migrated. The reviewer awarded 4 stars out of 5 A very interesting and much needed paper on policies regarding the physically challenged in medical schools. In my part of the world, the government mandates that a certain percentage of seats in medical schools are to be 'reserved' for this category of students. In my over two decades of teaching in medical schools here, I found that very few manage to get in. Why? we may ask. In the developing world, sad to say, some of these Physically challenged children are not given equal opportunities to study. There are few institutions which are Physically Challenged friendly. The social ostracism is present in ever so subtle ways so the parents don't guide them to professional courses. Many misconceptions abound among the semiliterate population that say the physically challenged are also mentally challenged. Let us hope that these biases will be corrected soon.

Reviewer Expertise:

NA

No decision status is available

# Reviewer report: Reviewer response for version 1

**DOI:** 10.21956/mep.19513.r28863

**Published:** 2018-03-25

**Authors:** Janusz Janczukowicz

**Affiliations:** 1 Medical University of Lodz

This review has been migrated. The reviewer awarded 5 stars out of 5 This paper addresses extremely important problems of medical students with disabilities. The analysis identifies interrelations of social norms and assumptions about disability with consecutive policies that most probably enhance the initial prejudice. While the general focus on patient safety is understandable, it should never be discriminating and obfuscating the importance of the inclusiveness of medical schools and the medical profession. The often unrecognised barriers to participation in education enhance the “otherness” and isolation of students with disabilities, with the resulting teaching presenting patients with disabilities as “the others”. This problem is correlated with general issues of doctors and medical students being afraid to admit own health problems and disabilities due to the lack of role models (e.g. teachers with disabilities).

Reviewer Expertise:

NA

No decision status is available

# Reviewer report: Reviewer response for version 1

**DOI:** 10.21956/mep.19513.r28861

**Published:** 2018-02-26

**Authors:** Petra Verdonk

**Affiliations:** 1 VU University Medical Center, dpt Medical Humanities

This review has been migrated. The reviewer awarded 4 stars out of 5 This is a very interesting policy analysis on a highly understudied ‘wicked problem’, namely the inclusion of medical students with disabilities in medical education. The authors analyze both UK and Australian policy documents which aim to assure quality of medical education (from Australian Medical Council 2010, Medical Board of Australia 2014, and General Medical Council 2015). The authors apply Bacchi’s six critical questions approach including ‘what presuppositions or assumptions underlie this representation of the problem’? In the policies, disability is framed as a potentional burden for medical schools, and schools ignore the possible ‘added value’ of an inclusive student population. For instance, peers could learn from their experiences, as they deconstruct the idea that categories of patients and doctors are mutually exclusive. Such policies and their concurrent practices are likely to silence students with disabilities. Hence, not only students themselves are disadvantaged by such practices, but also their peers and medical schools in general miss out on learning opportunities offered by bringing in students of diverse backgrounds and positions. The authors’ social constructivist approach is highly welcomed, interesting, and critical. At the ‘fixing the numbers’ level, this social constructivist analysis (problematization of reality) shows that practices rather focus on how to fix the students, to cater to ‘the special needs’ of students with disabilities. However at the ‘fixing the institutions’ level practices and procedures remain untouched, as the standards of competence are carved in stone and cannot be changed. A professional doctor is obviously an able-bodied doctor. Tragically so, such a medical model of disability disqualifies the student and locates the disability in the individual learner, not in the wider social and cultural context.

Reviewer Expertise:

NA

No decision status is available

# Reviewer report: Reviewer response for version 1

**DOI:** 10.21956/mep.19513.r28860

**Published:** 2018-02-26

**Authors:** Trevor Gibbs

**Affiliations:** 1 AMEE

This review has been migrated. The reviewer awarded 5 stars out of 5 Although anecdotal, I would like to begin this review with a personal reflection.Well over 10 years ago I was interviewing potential medical students for admission to a very highly respected school. The person to be interviewed was a male who had already been turned down by at least 3 , maybe 4, schools and this appeared to be his last chance. He has been born with phocomelia of his left arm and for most of his life had worn a mechanical device to replace his arm and hand. During the interview and much to the dismay of my co-interviewers ( I saw the worry in their eyes!) I asked him how he thought he would cope in the field of medicine. I said that I thought it might be a sensitive question and he didn't have to answer it if he did not wish to. He answered with enthusiasm, and said that this was the first time anyone had asked this question- he felt that in previous interviews, his interviewers had seen his prosthesis and assumed that medicine was not for him. His answer was relatively short- he felt that he had empathy with anyone with whatever disability and could understand them better, and medicine was advancing so quickly he knew that very soon there would be great advances in prosthetics and his new future arm would be almost as good as a real one! He was successful in getting to medical school , had no problems in completing the course, and had the intention of going into Primary Care post qualification. To me that summed up our attitude to disability and how people receive it through very different lenses.This paper I feel covered the subject very well, well constructed with excellent references- I learned much but was disappointed to also learn that we still have plenty to do. I fully recommend this paper to all those involved / interested in the area

Reviewer Expertise:

NA

No decision status is available
